# Effects of Blood Flow Restriction Training on Lower Extremity Strength, Power, and Athletic Performance: A Scoping Review

**DOI:** 10.7759/cureus.117499

**Published:** 2026-10-05

**Authors:** Aaron Jobi, Viswa Manidhara Raju Kurapati, Pradeep Vanguri

**Affiliations:** 1 Public Health, Nova Southeastern University, Fort Lauderdale, USA; 2 Biology, Nova Southeastern University, Fort Lauderdale, USA; 3 Health and Human Performance, Nova Southeastern University, Fort Lauderdale, USA

**Keywords:** blood flow restriction, lower-extremity, performance, power, strength

## Abstract

Blood flow restriction (BFR) training is increasingly being utilized as an alternative or supplement to traditional training methods to enhance performance adaptations while minimizing mechanical stress. The goal of BFR training is to reduce venous outflow while maintaining partial arterial inflow to the working muscle. This scoping review examines the relationship between BFR exercise modalities and lower extremity outputs such as strength, power, and explosive measures. A scoping review of 25 articles from the PubMed and ProQuest databases evaluated the effects of BFR on strength and hypertrophy, explosiveness, endurance/aerobic/sports-specific outcomes, and neuromuscular/vascular responses in athletic, healthy, and clinical populations. 44 of 77 reports sought for retrieval were not retrieved, limiting comprehensiveness of findings. Current findings indicate that BFR combined with low-load resistance training consistently improves lower extremity strength and muscle hypertrophy compared with low-load or non-BFR training in diverse populations. Effects are less consistent in some conditions, as published BFR research has not shown improved outcomes over rehabilitation approaches or prevented muscle loss during inactivity. BFR-based protocols may enhance post-activation enhancement, increase muscle activation and metabolic stress, and improve aerobic capacity, aerobic performance, and agility. However, its effects on maximal sprint performance and vascular responses remain inconsistent, with outcomes having high variation due to different pressure methods. Overall, the available literature suggests that BFR has been investigated across a broad range of lower extremity outcomes, with improvements more consistently reported for strength and hypertrophy, while findings for explosive performance, endurance, and vascular outcomes remain variable.

## Introduction and background

Blood flow restriction (BFR) training involves the application of external pressure to the proximal portion of a limb, partially restricting arterial inflow and limiting venous return during exercise [1]. It has gained increasing attention within sports performance, rehabilitation, and exercise physiology due to its ability to stimulate muscular adaptations while utilizing relatively low external loads. BFR training has been proposed as an alternative to traditional high-load resistance training, particularly in populations where heavy mechanical loading may not be viable or practical. 

Researchers have examined its application across a variety of exercise routines including resistance training, sprint exercise, walking protocols, warm-up activities, aerobic conditions, and sport-specific programs [2]. Previous studies have suggested that BFR training may improve lower extremity strength, muscle hypertrophy, neuromuscular activation, aerobic capacity, and explosive performance in both athletic and clinical populations [3]. BFR has frequently been used as an effective rehabilitation strategy for individuals who cannot tolerate heavy resistance exercise while still promoting meaningful strength and hypertrophy adaptations [4]. It has been incorporated into athletic settings because of its potential to enhance performance while minimizing mechanical stress on joints and connective tissue [5]. 

Despite the growing body of literature supporting BFR training protocols, findings across studies remain inconsistent. Variability in pressure, cuff width, exercise intensity, duration, participant populations, and outcome measures has contributed to debates regarding the effectiveness and safety of BFR interventions. Some investigations have demonstrated substantial improvements in strength and performance outcomes, whereas others have reported minimal differences compared with traditional training approaches. 

There remains variability regarding BFR training’s effectiveness across different lower extremity performance outcomes and populations. Lower-extremity outcomes such as strength, power, endurance, and neuromuscular adaptations are often examined independently rather than synthesized collectively. The purpose of this scoping review is to map the current literature examining the effects of BFR on lower extremity muscular strength, explosive power, endurance capacity, and neuromuscular adaptations across athletic, physically active, healthy, older adult, and clinical populations. 

## Review

Methodology

A scoping review of peer-reviewed published articles was conducted and reported in accordance with the Preferred Reporting Items for Systematic Reviews and Meta-Analyses Extension (PRISMA-ScR) guidelines [6]. The purpose of the review was to examine the current literature on the effects of BFR training on lower extremity strength, power, and athletic performance in athletic, physically active, healthy, older, and clinical populations. 

The broad scope of this review style was selected because the available literature encompasses diverse participant populations, exercise protocols, BFR application methods, and outcome measures. 

Search strategy 

A literature search was conducted using PubMed and ProQuest from January 2026 to May 2026. These databases were chosen based on their coverage of biomedical, health sciences, exercise science, and peer-reviewed literature relevant to BFR training. The search included studies published from January 2011 to January 2026. The search strategy included the Boolean expression ("blood flow restriction") AND ("power" OR "sprint" OR "lower extremity"). Two reviewers independently identified a total of 207 articles initially: 172 from PubMed and 35 from ProQuest. Duplicate screening was performed, and 5 duplicate records were identified. 21 articles were excluded as they were not published within the past 15 years, reducing the total number of usable articles to 181. The 181 unique articles subsequently underwent title and abstract screening, performed by both reviewers, further reducing the total number of articles to 77. However, 44 articles were not accessible, leading to 33 reports assessed against the inclusion criteria. 

Inclusion criteria were as follows: studies must be based on BFR’s impact on lower extremity power, strength, explosiveness, and/or muscle activation, written in English, and with full-text availability. The studies must employ a BFR training or warm-up protocol with athletes or physically active humans as subjects. Participant characteristics, including age, sex, training status, and clinical status, were considered during study selection and data extraction. No restrictions were imposed on participant sex. Participant age was not used as an exclusion criterion, allowing the review to capture evidence across young adults, older adults, athletic populations, and clinical populations. The included literature represented a range of participant populations, including young adults, athletes, older adults, and clinical populations. Studies involving participants with underlying conditions were included when they met the predefined BFR and lower extremity outcome criteria. Studies involving conditions such as knee osteoarthritis, total knee arthroplasty, immobilization, bed rest, or disuse were retained when they met the intervention and outcome criteria. Exclusion criteria included non-primary articles or reviews, articles regarding BFR’s impact on upper extremity outcomes, or articles that lacked adequate findings with synthesizable data. Following title, abstract, and full-text screening, performed by both reviewers independently using the predefined inclusion and exclusion criteria, eight articles were excluded (upper extremity related, *n* = 1; unrelated/inadequate outcomes or measures, *n* = 3; reviews and non-primary articles, *n* = 4), resulting in 25 studies included in the final qualitative synthesis, as presented in Figure 1. Disagreements between the reviewers were resolved through discussion and consensus.

**Figure 1:**
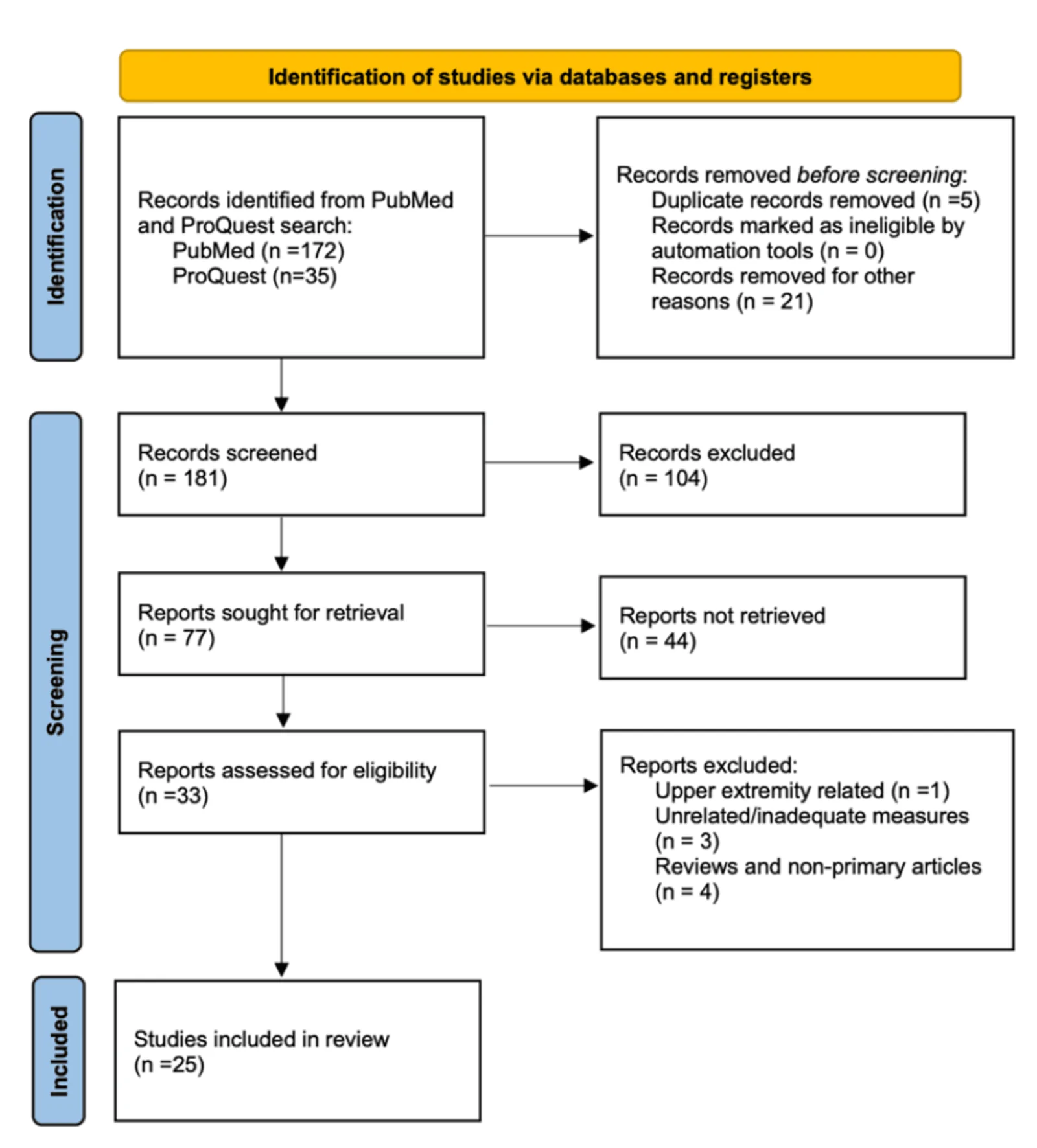
PRISMA-ScR diagram showing study identification, screening, and inclusion/exclusion. PRISMA-ScR, Preferred Reporting Items for Systematic Reviews and Meta-Analyses extension for Scoping Reviews

The study selection process was conducted by two reviewers independently using the predefined eligibility criteria. Following identification and screening, eligible studies were charted according to study characteristics, participant population, BFR intervention characteristics, exercise protocol, and reported lower extremity outcomes. Due to the variation in BFR protocols, cuff pressures, and outcome measures, a qualitative synthesis was performed instead of a quantitative meta-analysis. The included studies were subsequently organized into thematic categories based on the primary outcomes investigated, including strength and hypertrophy, explosive performance, endurance and sport-specific conditioning, and neuromuscular/vascular responses, with the characteristics and primary findings of the included studies summarized in Tables 1-4. 

**Table 1:** Characteristics, intervention protocols, and outcomes of studies relating to BFR's impact on strength and hypertrophy adaptations. BFR, blood flow restriction; 1RM, one-repetition maximum; EMS, electrical muscle stimulation; VL, vastus lateralis; MVC, maximal voluntary contraction; TKA, total knee arthroplasty

Author(s), year	Design	Sample and population	Occlusion method/pressure	BFR protocol and duration	Outcomes assessed	Main outcome data
Bowman et al., 2019 [7]	Randomized controlled trial	*n* = 26 healthy adults	Cuff on proximal thigh; 80% arterial occlusion pressure	Low-load resistance exercise at 30% 1RM; 30/15/15/15 reps; 2×/week for 6 weeks	Knee-extension strength, total work, average power, thigh/leg circumference, contralateral effects	Knee-extension peak torque +11% vs. +3% control; total work +15% vs. +6%; average power +12% vs. +4%; thigh circumference +3.5% vs. +0.8%.
Kang et al., 2015 [8]	Randomized controlled trial	*n* = 17 college students	Cuff/wrap applied to proximal thigh; pressure reported according to study protocol.	Bodyweight squats and lunges with BFR; 3×/week for 6 weeks	Isokinetic knee flexor/extensor function; thigh circumference	Knee-flexor peak torque increased 20.2±15.2% vs. 11.0±18.7% control; thigh circumference also significantly increased.
Jia et al., 2025 [9]	Controlled intervention	*n* = 55 young adults	Individualized pressure according to protocol	Low-intensity resistance exercise combined with BFR; lower-extremity training performed over a 12-week intervention	Lean body mass, lower-extremity strength, sports biomechanics	Significant increases in lean body mass, 1RM, and knee isometric strength (*P *< 0.05)
Patterson et al., 2011 [10]	Within-subject controlled training study	*n* = 10 untrained individuals aged 62-73 years	Cuff positioned above knee	Low-load plantar-flexion resistance training; 25% 1RM; 4 wk	1RM, maximum voluntary contraction, isokinetic strength, calf blood flow	BFR produced greater improvements in 1RM, maximum voluntary contraction, and isokinetic strength than unrestricted low-load training; post-occlusive calf blood flow also increased.
Sakamaki et al., 2011 [11]	Controlled intervention	*n* = 17 young men	Cuffs placed around proximal thighs	Walking at 50 meters/minute, 5 × 2-min bouts; 2×/day, 6 days/week for 3 weeks	Upper- and lower-leg muscle volume; trunk muscle volume	Upper-leg muscle volume increased 3.8% and lower-leg volume 3.2% with BFR walking; no significant trunk changes.
Yang et al., 2025 [12]	Randomized controlled trial	*n* = 60 male university football players with knee OA	Proximal thigh cuff; pressure based on thigh circumference: 200-350 mmHg	Squats at 25% 1RM, 3 sets × 30 reps; 4×/week for 8 weeks; BFR ± electrical stimulation	Peak torque, sprinting, countermovement jump, agility, muscle activation, rectus femoris cross-sectional area	BFR improved strength, sprinting, countermovement jump, agility, muscle activation, and muscle cross-sectional area; combined BFR+EMS produced larger changes in several outcomes.
Gavanda et al., 2020 [13]	Randomized matched-pair controlled trial	*n* = 21 healthy males	7-cm cuffs below patella	Calf raises at 30% 1RM, 4 sets to failure; 2×/week for 6 weeks	Calf volume, gastrocnemius thickness, leg stiffness, 1RM, pain	No significant group differences for calf volume, thickness, stiffness, or 1RM. Muscle thickness increased by 0.07 cm vs. 0.04 cm; 1RM increased by 40 vs. 34 kg. BFR required fewer reps (52 vs. 70/session).
Fuchs et al., 2025 [14]	Randomized within-subject bed-rest study	*n* = 12 healthy young men	Proximal thigh cuff; 200 mmHg; control/sham 10 mmHg	Passive BFR 3×/day, each session 3 × 5-min occlusions; 14 days	Lean body mass, thigh volume, 1RM leg extension, muscle protein synthesis	Lean body mass decreased by 1.8 ± 1.0 kg. Thigh volume decreased from 7.0 to 6.7 L in the BFR group and from 7.1 to 6.7 L in the control group, with no treatment difference (P = 0.497). 1RM also declined, with no benefit from BFR (*P* = 0.594).
Slysz et al., 2021 [15]	Randomized controlled trial	*n* = 30 healthy adults	Lower-extremity BFR during disuse; repeated occlusion cycles	BFR 3 × 5-min cycles; BFR ± EMS during unloading; 14 days	Thigh lean mass, VL thickness, knee-extension MVC	Thigh lean mass decreased 4% in control, 3% in BFR, 0.3% in BFR+EMS. MVC decreased 18%, 10%, and 18%, respectively; no significant between-group strength difference.
Majors et al., 2022 [16]	Retrospective comparative study	*n* = 48 patients with painful TKA	50%-80% limb occlusion pressure	20%-30% of 1-repetition maximum; 4 × 30/15/15/15 repetitions; open- and closed-chain lower-extremity exercises; 12-15 sessions over 6-10 weeks	Power per repetition, peak torque, and work per repetition for knee flexion and extension in affected and contralateral legs	Both groups improved strength; no significant difference between blood flow restriction and standard physical therapy

**Table 2:** Characteristics, intervention protocols, and outcomes of studies relating to BFR's impact on power and post-activation performance. BFR, blood flow restriction

Author(s), year	Design	Sample and population	Occlusion method/pressure	BFR protocol and duration	Outcomes assessed	Main outcome data
Doma et al., 2020 [17]	Randomized crossover	*n* = 18 trained men	130% systolic blood pressure	Body-weight single-leg lunges, 3 × 8 repetitions; familiarization in week 1, experimental sessions in weeks 2-3; jump performance assessed immediately and 3, 6, 9, 12, and 15 minutes after exercise	Drop-jump height, flight time, peak power, and reactive strength	Blood flow restriction lunges increased jump height, flight time, and power during the 6-15-minute recovery period.
Gepfert et al., 2020 [18]	Randomized crossover	*n* = 10 judo athletes	Intermittent external compression at 100% or 150% arterial occlusion pressure	Back squats at 70% one-repetition maximum, 3 × 3 repetitions	Peak power, mean power, peak bar velocity, and mean bar velocity	150% arterial occlusion pressure significantly increased all power and velocity measures compared with control and 100% pressure; 100% pressure produced no significant improvement.
Sun et al., 2023 [19]	Repeated-measures study	*n* = 12 elite female football players	50%, 60%, or 70% arterial occlusion pressure	Semi-squats at 30% one-repetition maximum, 4 × 30/15/15/15 repetitions; 60-s rest between sets; acute testing at 15 seconds, 5, 10, and 15 minutes after exercise	Countermovement jump height, squat jump height, lower-limb muscle activation, and perceived exertion	50% and 60% arterial occlusion pressure significantly improved jump height, peak power, and rate of force development; 50% produced the strongest overall post-exercise response.
Wei et al., 2025 [20]	Randomized controlled trial	*n* = 16 male collegiate volleyball players	Approximately 60% arterial occlusion pressure (~200 mmHg)	Barbell squats at 30% one-repetition maximum, 2 × 30 repetitions; acute testing at 15 s, 4, 8, 12, 16, and 20 minutes after exercise	Countermovement jump height, peak power, vertical ground reaction force, rate of force development, and muscle electrical activity	Blood flow restriction training significantly increased countermovement jump height from 4 to 20 minutes, with a significant between-group advantage at 16 minutes; peak power also favored blood flow restriction at several time points.
Zhou et al., 2024 [21]	Randomized controlled trial	*n* = 26 healthy male university athletes	Upper-thigh cuffs; 200 mmHg during weeks 1-4 and 220 mmHg during weeks 5-8	Complex training 3 times/week for 8 weeks; squats, split squats, and deadlifts at 20-30% one-repetition maximum, paired with jumps; 3 sets with 15-20 resistance repetitions + 6-8 jumps.	Squat one-repetition maximum, squat jump, countermovement jump, mean and peak power, and mean and peak bar velocity.	Blood flow restriction complex training produced greater improvements in power output and bar velocity, while strength and jump-height improvements were comparable with conventional complex training.
Jiang et al., 2025 [22]	Randomized controlled trial	*n* = 24 adolescent female wrestlers	Upper-thigh cuff; 180 mmHg inflation pressure	Low-intensity half-squats at 30% one-repetition maximum, 4 sets of 30/15/15/15 repetitions, 60-s rest; combined group also performed high-intensity half-squats at 75% one-repetition maximum; 3 sessions/week for 6 weeks.	Knee peak torque, squat jump height, countermovement jump height, and peak rate of force development	Combined low- and high-intensity blood flow restriction training increased right knee extension peak torque 10.1%, squat jump height 13.2%, countermovement jump height 9.7%, and peak rate of force development 9.2% and 8.0%, respectively.
Zhang et al., 2025 [23]	Repeated-measures study	*n* = 15 healthy male university students	60% arterial occlusion pressure	Four acute warm-up conditions: low-intensity exercise at 30% one-repetition maximum and high-intensity exercise at 70% one-repetition maximum; back squats 3 × 4 repetitions, 30-second rest; testing immediately and 3, 6, 9, and 12 minutes post-exercise	Knee extension torque, muscle electrical activity, countermovement jump flight height, and peak power	High-intensity blood flow restriction produced greater knee extension torque at several time points, while low-intensity blood flow restriction improved jump height at 3 and 6 minutes; responses varied according to exercise intensity.

**Table 3:** Characteristics, intervention protocols, and outcomes of studies relating to BFR's impact on endurance, aerobic, and sport-specific conditioning adaptations. BFR, blood flow restriction; 1RM, one-repetition maximum; VO_2_max, maximal oxygen uptake; VO_2_peak, peak oxygen uptake; HIIT, high-intensity interval training; MIIT, moderate-intensity interval training

Author(s), year	Design	Sample and population	Occlusion method/pressure	BFR protocol and duration	Outcomes assessed	Main outcome data
Held et al., 2024 [24]	Controlled intervention study	*n* = 11 highly elite male rowers; age 22.1 ± 1.6 years	Customized elastic wraps applied to lower limbs	Low-intensity rowing (< 2 mmol/L) with BFR; 3 ×/week; 2 × 10-minute occlusion periods/session; 5 weeks	VO₂max, peak power output, 2,000-m rowing performance	VO₂max increased by 0.30 L/min; peak power output increased by 14 W, while 2,000-m rowing performance showed no noticeable improvement.
Chen et al., 2025 [25]	Randomized controlled trial	*n* = 33 male college basketball players; BFR-MIIT, *n* = 17; HIIT, *n* = 16	KAATSU devices applied to lower extremities; 180-200 mmHg	Moderate-intensity interval training at 70%-75% of maximum heart rate with BFR; 4 × 4 training protocol; 2 ×/week for 6 weeks	VO₂max, Wingate peak power, jumping ability, 30-m sprint, change-of-direction ability	VO₂max increased from 39.48 ± 6.22 to 44.48 ± 4.77 mL/kg/minutes; significant improvements occurred in countermovement, squat, and drop jumps and 505 change-of-direction performance; anaerobic peak power did not significantly improve.
Liu et al., 2025 [26]	Randomized controlled trial	*n* = 24 well-trained male collegiate basketball players; age 21.0 ± 1.6 years	Lower-extremity BFR at 100-130% of leg systolic pressure	3-vs-3 basketball small-sided games; 4 sets × 3-4.5-minute bouts with 3-minute rest periods; 8 sessions over 4 weeks	Peak aerobic power, VO₂peak, Wingate performance, back-squat 1RM, countermovement jump, agility, 30-m sprint, repeated-sprint ability	Peak aerobic power increased 9.5% with BFR vs. 4.5% control; Wingate mean power increased 4.6%, and countermovement jump height increased 6.8%; VO₂ peak increased 6.4% with BFR vs. 4.4% control.
Chen et al., 2021 [27]	Counterbalanced within-subject experimental study	*n* = 12 male sprinters	BFR during running; occlusion pressure 1.3 × resting systolic blood pressure	Running at 50% heart rate reserve; 5 × 2-minute bouts with 1-minute rest periods; acute warm-up intervention	Isokinetic knee flexor/extensor strength, hamstring-to-quadriceps ratio, 60-m sprint, muscle activation, blood lactate, heart rate, perceived exertion	BFR significantly increased knee-flexor strength and hamstring-to-quadriceps ratio and produced greater muscle activation, heart rate, perceived exertion, and blood lactate; no significant improvement occurred in knee-extensor strength or 60-m sprint performance.
Rojas et al., 2024 [28]	Randomized crossover study	*n* = 15 men	Continuous or intermittent lower-extremity BFR	2 × 30-second maximal cycle sprints at resistance equal to 7.5% body mass; 2-minute rest; acute intervention	Concentric peak torque, maximal voluntary isometric contraction torque, muscle thickness, surface electromyography, sprint revolutions per minute, power output	Peak torque decreased from 220.3 ± 47.6 to 147.9 ± 27.7 Nm and maximal voluntary isometric contraction decreased from 355.1 ± 72.5 to 252.2 ± 45.5 Nm; muscle thickness increased from 1.77 ± 0.31 to 1.96 ± 0.30 cm. Neuromuscular fatigue and sprint performance were generally comparable across BFR and no-BFR conditions.

**Table 4:** Characteristics, intervention protocols, and outcomes of studies relating to BFR's impact on neuromuscular, physiological, vascular, and safety responses. BFR, blood flow restriction; AOP, arterial occlusion pressure; PWV, pulse wave velocity; 1RM, one-repetition maximum

Author(s), year	Design	Sample and population	Occlusion method/pressure	BFR protocol and duration	Outcomes assessed	Main outcome data
Zhuan et al., 2024 [29]	Randomized self-controlled crossover study	*n* = 12 male university students; age 23 ± 2 years; ≥ 3 years resistance-training experience	TheraBand cuffs at proximal thigh; continuous 40% AOP, intermittent 50% AOP, or intermittent 60% AOP	Squats at 75% 1RM; 3 sets × 8 reps with 2-minute rest; four acute conditions separated by 72 hours	Lower-limb and core muscle activation, thigh circumference, perceived exertion	All BFR modes increased anterior thigh muscle activation compared with control during the first two sets. Continuous 40% AOP produced more consistent posterior-thigh activation; intermittent 60% AOP increased erector spinae activation. BFR did not further increase thigh circumference and generally increased perceived exertion.
Rolnick et al., 2024 [30]	Randomized crossover study	*n* = 20 physically active adults; age 23 ± 5 years; 7 females	Delfi Personalized Tourniquet System; 60% of supine limb occlusion pressure; autoregulated and non-autoregulated BFR	Dumbbell wall squats at 20% 1RM; 4 sets to volitional fatigue; acute intervention	Central and peripheral arterial stiffness, carotid-femoral PWV, carotid-radial PWV, blood pressure	Carotid-femoral PWV increased after non-autoregulated BFR and no-BFR exercise but not after autoregulated BFR. Carotid-radial PWV increased only after no-BFR exercise; the carotid-femoral PWV response favored autoregulated over non-autoregulated BFR.
Fahs et al., 2014 [31]	Within-subject controlled training study	*n* = 16 middle-aged adults; 11 men (55 ± 8 years) and 5 postmenopausal women (57 ± 5 years)	One leg trained with BFR and the contralateral leg without BFR	Unilateral low-load knee-extension exercise to fatigue with and without BFR; 6 weeks	Femoral PWV, calf blood flow, calf vascular conductance, calf venous compliance	Femoral PWV increased slightly following training. Calf blood flow increased in the non-BFR limb but did not change in the BFR limb; calf venous compliance did not change in either limb.

Results 

Strength and Hypertrophy Adaptations 

Studies examining the effects of BFR training on muscular strength and hypertrophy generally reported positive adaptations across a variety of populations and protocols. Improvements were observed in muscle strength, peak torque, power output, and muscle cross-sectional area when BFR was combined with low-load resistance exercise, bodyweight training, or electrical stimulation [7-13]. These benefits were reported in healthy young adults, older adults, athletes, and individuals undergoing rehabilitation [7-13]. However, others reported that BFR alone was insufficient to prevent losses in muscle mass and strength during periods of prolonged inactivity or bed rest [14,15]. Additionally, the literature was not uniformly positive, as some studies found outcomes similar to traditional training methods [16]. These findings demonstrate variation in reported effects across intervention types and populations. The studies included in this review and their primary findings are summarized in Table 1. 

Explosive Performance and Post-activation Performance Enhancement

Studies investigating the effects of BFR training on explosive performance and post-activation performance enhancement (PAPE) reported favorable outcomes in athletes and physically active individuals [17-23]. BFR applied during conditioning activities including lunges, semi-squats, resistance exercises, and complex training was associated with acute improvements in jump performance, power output, bar velocity, force development, and muscle activation during the recovery period following exercise [17-23]. Studies found that the use of BFR protocols contributed to a greater improvement in vertical jump height and explosive lower limb performance when compared to traditional exercise protocols, particularly when individualized or moderate arterial occlusion pressures were used [19,20,22]. Additionally, BFR combined with high-intensity training was associated with greater improvements in power-related measures and force production than non-BFR conditions [18,21,23]. However, some outcomes using BFR protocols such as maximal strength and jump height, were not consistently better compared to traditional methods despite improvements in power output and movement velocity [21]. The studies included in this review and their primary findings are summarized in Table 2.

Endurance, Aerobic, and Sport-Specific Conditioning Adaptations

The effects of BFR on endurance, aerobic fitness, and sport-specific conditioning generally reported positive physiological and performance adaptations across athletic populations [24-28]. BFR combined with low- to moderate-intensity aerobic and interval-based exercise was associated with improvements in aerobic capacity including increases in VO_2_max, aerobic power, and anaerobic performance measures [24-26]. Additionally, incorporating BFR into sport-specific activities such as small-sided basketball games and interval training enhanced countermovement jump performance, repeated sprint ability, agility, and lower extremity functional outcomes [25,26]. Studies also reported increased muscle activation and physiological stress responses during exercise performed with BFR, suggesting a greater training stimulus despite lower external workloads [27,28]. However, improvements in maximal sprint performance and sport-specific performance outcomes were not consistently observed [24,27,28]. The studies included in this review and their primary findings are summarized in Table 3. 

Neuromuscular, Physiological, Vascular, and Safety Responses

Studies investigating the neuromuscular, physiological, vascular, and safety responses to BFR training suggest that BFR can enhance muscle activation and metabolic stress while producing variable vascular adaptations [29-31]. High-intensity exercise performed with BFR was associated with greater lower limb muscle activation and increased metabolic demands compared with traditional exercise conditions [29]. Different pressure levels appear to influence the physiological effects of BFR by producing distinct muscle recruitment patterns and vascular responses [29,30]. Individualized/auto-regulated occlusion has been associated with more favorable vascular outcomes and reduced adverse vascular responses compared with fixed-pressure methods [30,31]. The studies included in this review and their primary findings are summarized in Table 4.

Discussion 

This scoping review examines available literature relating to the effects of BFR training on lower extremity strength, hypertrophy, explosive performance, endurance capacity, and neuromuscular and vascular responses. Across the included studies, BFR was most consistently associated with improvements in muscular strength and hypertrophy when applied during low-load resistance training, while findings related to explosive performance, endurance outcomes, and vascular adaptations were more variable and context-dependent.

Strength and Hypertrophy Adaptations 

Multiple studies identified in this scoping review reported improvements in lower extremity strength and muscle hypertrophy. Across multiple randomized and controlled trials, low-load resistance training combined with BFR produces greater increases in knee extension strength, torque, and muscle size compared with low-load or non-BFR conditions [7,8,10,11]. This mixed training method can also yield similar results to conventional training methods, but with a lower amount of repetitions [13]. This indicates a more time-efficient method of training. These findings suggest that BFR can partially compensate for the reduced mechanical loading typically required for traditional hypertrophy and strength adaptation processes.

Importantly, these adaptations were observed across diverse populations, including young adults, trained individuals, and older adults, indicating that the hypertrophic and strength-enhancing effects of BFR are not limited to a specific demographic [9,10,13]. Additionally, combined interventions such as BFR with electrical stimulation or resistance training appear to further augment muscle cross-sectional area and force production, particularly in clinical or sports-specific populations [12,15]. However, the magnitude of benefit appears reduced in clinical populations, as shown in post-total knee arthroplasty rehabilitation, where BFR did not produce superior strength gains compared with standard physical therapy [16]. Furthermore, passive BFR without exercise failed to prevent muscle atrophy during bed rest, highlighting that active muscular contraction remains essential for meaningful adaptation [14]. Overall, improvements were most consistently reported when combined with active low-load resistance exercise rather than as a standalone intervention.

Explosive Performance and PAPE 

Evidence regarding explosive performance and PAPE indicates that BFR acutely enhances power-related outcomes, particularly in jump and sprint-based tasks. Several studies demonstrated improvements in countermovement jump height, drop jump performance, peak power output, and bar velocity following BFR-based conditioning or resistance protocols [17-20,22]. These acute enhancements appear to be linked to increased neuromuscular activation and metabolic stress, which may facilitate temporary improvements in rate of force development in the lower extremities. 

However, the effects of BFR on explosive performance are not uniform. While moderate occlusion pressures tend to produce the greatest improvements in jump performance, excessively high or low pressures may actually reduce effectiveness due to excessive fatigue or insufficient stimulus [19]. High-intensity BFR appears to enhance maximal power output, while low-intensity BFR may produce greater neuromuscular activation without consistent improvements in performance outcomes [23]. These findings suggest that performance responses are highly sensitive to both training intensity and occlusion pressure.

Despite consistent acute improvements, long-term superiority over traditional resistance training is not well established. Some studies report no additional benefit of BFR for jump height or maximal strength beyond traditional training methods [21]. This indicates that BFR may be most effective for acute improvements rather than chronic enhancement of explosive performance.

Endurance, Anaerobic, and Sport-Specific Adaptations 

BFR also appears to contribute to improvements in aerobic and sports-specific performance, although findings are more mixed compared with strength outcomes. Improvements in VO_2_max have been observed in elite endurance athletes following low-intensity BFR training, suggesting that BFR may stimulate aerobic adaptations even under reduced mechanical load conditions [24]. Moderate intensity interval training combined with BFR has been shown to improve aerobic capacity, anaerobic performance, and lower extremity functional outcomes compared with interval training alone [25]. This suggests that BFR may serve as potential supplemental tool during endurance workouts.

In the context of team sports, incorporating BFR into small-sided games has been associated with improvements in repeated sprint ability, countermovement jump performance, and change of direction speed [26]. However, not all sports-specific applications demonstrate performance transfer. BFR combined with running or sprint interval training increases physiological stress and muscle activation, but does not consistently improve sprint performance [27,28]. 

These findings suggest that BFR may enhance metabolic conditioning and sub-maximal performance capacity, but translation to maximal performance capacity remains inconsistent and likely depends on training specificity and personalization.

Neuromuscular and Vascular Responses 

BFR consistently increases muscle activation and metabolic stress during exercise. High-intensity exercise combined with BFR has been shown to elevate activation of both anterior and posterior thigh muscles, supporting enhanced motor unit recruitment under restricted conditions [29]. These neuromuscular responses may lead to the observed improvements in strength and power-related outcomes. 

Vascular responses to BFR are less consistent and raise potential safety issues. Although personalized BFR training appears to alleviate acute increases in arterial stiffness compared with non-individualized methods, evidence suggests that BFR may still induce small increases in arterial stiffness following resistance training [30,31]. These findings highlight the importance of personalized occlusion pressure and device selection to optimize safety and vascular outcomes.

Additionally, evidence from disuse models indicates that BFR alone is insufficient to prevent muscle atrophy or strength loss during inactivity [14]. Combining BFR with electrical stimulation may partially mitigate muscle loss during short-term inactivity, although it does not fully preserve strength [15]. This further supports the role of BFR as a supplement during exercise rather than a standalone intervention.

Practical Implications 

Overall, BFR training appears to be an effective supplemental method for improving lower extremity strength and hypertrophy, particularly when combined with low-load resistance exercise to reduce mechanical loading stress. It may also provide acute benefits for explosive performance through PAP-related mechanisms and contribute to aerobic and anaerobic conditioning adaptations in sport-specific settings. Its effectiveness is highly dependent on application variables, including occlusion pressure, training intensity, and exercise protocol. 

Moderate occlusion pressures and active resistance-based protocols appear most effective. Excessive pressure or passive application may limit benefits or increase fatigue. Coaches and clinicians should therefore consider individualization of BFR training to optimize performance outcomes while minimizing adverse vascular responses.

Limitations and Future Directions 

The scoping review has several limitations. The literature search only included studies from PubMed and ProQuest. While these databases provide substantial coverage of BFR studies, relevant studies indexed in other databases may have not been identified. Additionally, 77 reports were sought for retrieval, but 44 were not retrieved, limiting the conclusions drawn through this review. Variability across study populations, training protocols, occlusion pressures, and outcome measures limit direct comparability between studies. Most included studies involved acute or short-term interventions, limiting conclusions regarding long-term adaptations. Future research should prioritize standardized BFR protocols, longer intervention durations, and direct comparisons with traditional high-load resistance training across both athletic and clinical populations.

## Conclusions

BFR training appears to be an effective supplemental method for improving lower extremity strength, hypertrophy, and performance outcomes in athletic, active, and clinical populations. The strongest evidence supporting BFR comes from studies in which it is integrated with active low-load resistance exercise, as several studies documented improvements in muscle strength, torque, and hypertrophy while using lower external loads. BFR may also provide acute benefits regarding explosive performance and PAPE, particularly in jump and power-based tasks; however, these outcomes also appear to be influenced by occlusion pressure, exercise intensity, and recovery. Endurance and sport-specific outcomes were more variable, with some studies showing improvements in VO_2_max, repeated sprint ability, and aerobic or anaerobic performance, while others found limited transfer to maximal sprint performance. BFR did not consistently provide benefits to certain populations, such as those with total knee arthroplasty, where BFR did not provide significant improvements compared to the control group. Regardless, BFR appears to serve as a useful supplemental training strategy for rehabilitation and athletic performance, but future research could focus on establishing standardized protocols, individualized cuff pressures, and long-term outcomes. 
